# Antiproliferation of berberine is mediated by epigenetic modification of constitutive androstane receptor (CAR) metabolic pathway in hepatoma cells

**DOI:** 10.1038/srep28116

**Published:** 2016-06-17

**Authors:** Lei Zhang, Xiao-Jie Miao, Xin Wang, Hai-Hui Pan, Pu Li, Hong Ren, Yong-Rui Jia, Chuang Lu, Hong-Bing Wang, Lan Yuan, Guo-Liang Zhang

**Affiliations:** 1 Department of Pharmacology, School of Basic Medical Sciences, Peking University, Beijing, 100191, China; 2 Medical and Healthy Analytical Center, Peking University, Beijing, 100191, China; 3 Department of Drug Metabolism & Pharmacokinetics, Biogen, Cambridge, Massachusetts, USA; 4 Department of Pharmaceutical Sciences, School of Pharmacy, University of Maryland, Baltimore, USA

## Abstract

Constitutive androstane receptor (CAR) regulates hepatic xenobiotic and energy metabolism, as well as promotes cell growth and hepatocarcinogenesis. Berberine is an ancient multipotent alkaloid drug which derived from *Coptis chinensis* plants. Here we report that berberine is able to be cellular uptake and accessible to chromatin in human hepatoma HepG2 cells. Berberine induces more apoptosis, cell cycle arrest, but less ROS production in CAR overexpressed mCAR-HepG2 cells. Moreover, berberine inhibits expressions of *CAR* and its target genes *CYP2B6* and *CYP3A4*. Furthermore, berberine enhances DNA methylation level in whole genome but reduces that in promoter regions CpG sites of *CYP2B6* and *CYP3A4* genes under the presence of CAR condition. These results indicated that the antiproliferation of berberine might be mediated by the unique epigenetic modifying mechanism of CAR metabolic pathway, suggesting that berberine is a promising candidate in anticancer adjuvant chemotherapy, due to its distinct pharmacological properties in clinic.

Constitutive androstane receptor (CAR) is a transcription factor which plays an important role in the hepatic exogenous and endogenous metabolisms by regulating the expression of its target genes^1^,^2^. In the presence of activators such as phenobarbital (PB), CAR is dissociated from its co-chaperone partners, and translocate into the nucleus^3^,^4^. Following the dimerizing with the retinoid X receptor (RXR) then binding to PB responsive enhancer modules (PBREM) in its target promoters, the expressions of many metabolic target genes are transcriptional activated^5^. CAR regulates numerous genes encoding drug- and xenobiotic- metabolizing enzymes, such as cytochrome P450 2B6 (CYP2B6) and CYP3A4 isoenzymes^6^. CAR also influences endobiotic energy metabolism, including lipogenesis, fatty acids oxidation and glucose homeostasis^7^. Recently research showed that CAR promotes multiple tumor proliferation and metastasis, and induces resistance for antitumor chemotherapeutics^8^,^9^,^10^. Therefore, targeting CAR metabolic pathway has been considered a novel anticancer drug development approach.

Berberine is an isoquinoline alkaloid derived from *Coptis chinensis* plants^11^. Berberine-containing plants (Huanglian) have been used to treat diarrhea, gastroenteritis, feverous and hepatic disorders in traditional Chinese medicines for centuries^12^,^13^. Pharmacological effects of berberine including anti-inflammatory, hypoglycemic, hypolipidemic and neuroprotection have been well demonstrated^14^,^15^,^16^. As an over the counter (OTC) medicine in China with low reported side effect, berberine is also widely used in clinic to treat dysentery, type 2 diabetes mellitus, hypertension, hypercholesterolemia and neurodegenerative disorders^17^. Recent studies have proved that berberine can inhibits proliferation of multiple human tumor cells^18^,^19^. However, whether the antitumor effect of berberine is associated with CAR metabolizing pathway remains unclear.

DNA methylation is one of the most common epigenetic modification mechanisms in gene expression, which often exhibits as changes of 5-methylcytosine level in CpG dinucleotide (CpG-island) located in the promoters of many genes or chromatins^20^. Abnormal DNA methylation including global genome hypomethylation is caused by oncogenes activation and chromosomal instability, while hypermethylation in GpG-rich promoter regions results in silencing of tumor suppressor genes in hepatocarcinogenesis^21^,^22^,^23^,^24^,^25^. The biotransformation and disposition of berberine is mainly oxidative demethylation and demethylenation by hepatic phase I drug metabolic CYP450 isoenzyme, and subsequent glucuronidation to facilitate excretion^26^,^27^,^28^. The metabolites of berberine are remained the pharmacological activities in spite of potency. However, the pharmacological mechanism of berberine such as whether it involves epigenetic modification of CAR and the target genes CYP2B6 and CYP3A4 metabolic pathway remains unknown. The current research investigated whether berberine could be intracellular uptake and accessible to chromatin, inhibit proliferation, and influence DNA methylation states of CAR and its target gene *CYP2B6* and *CYP3A4* pathway in human hepatoma HepG2 cells.

## Results and Discussion

### Intracellular distribution and anti-proliferation of berberine in HepG2 cells

**Figure 1:**
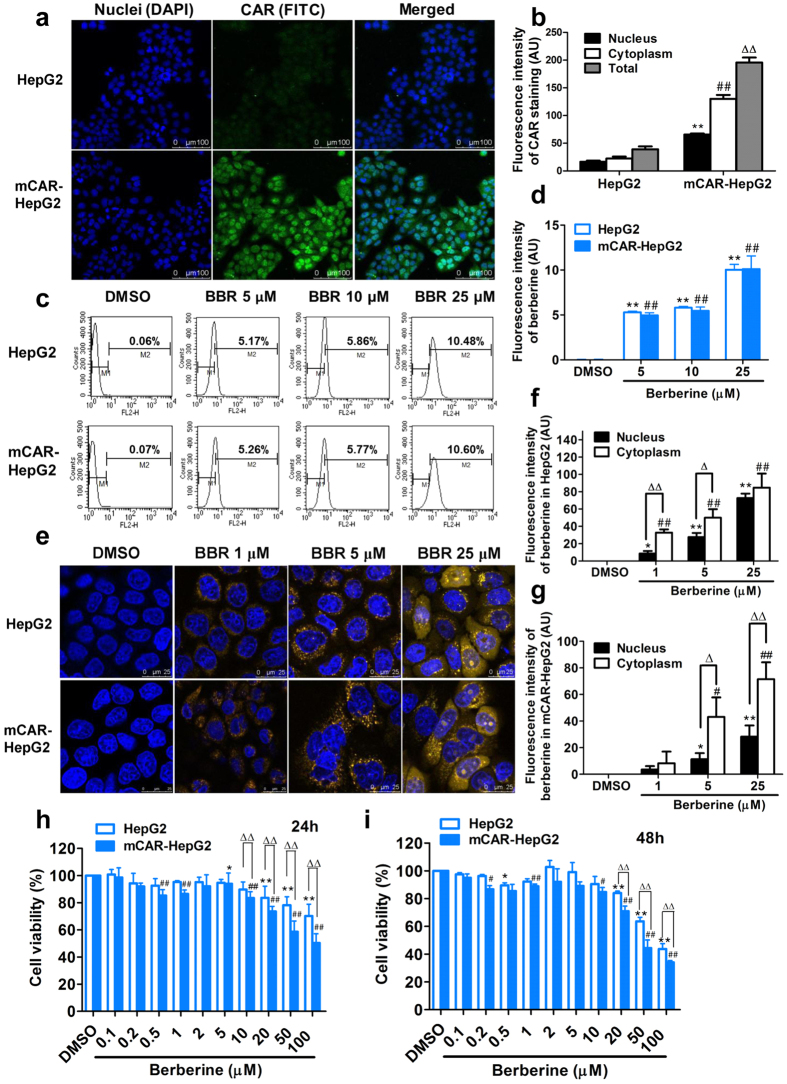
Berberine is able to be cellular uptake, accessible to chromatin and inhibit proliferation in hepatoma HepG2 cells. (**a,b**): Visual distribution (**a**) and quantitative analysis (**b**) of constitutive androstane receptor (CAR) in hepatoma HepG2 and mCAR-HepG2 cells were detected by confocal laser scanning microscopy (CLSM). (c and d): Intracellular uptake (**c**) and quantitative analysis (**d**) of berberine (BBR, 5, 10, 25 μM) in HepG2 and mCAR-HepG2 cells were measured by flow cytometry. (**e–g**): Visual intracellular localization (**e**) and quantitative analysis of fluorescent berberine (BBR, 1, 5, 25 μM) were observed in HepG2 cells (**f**) and mCAR-HepG2 cells (**g**) by CLSM. (h and i): Effects of berberine (0.1–100 μM) on the viability/proliferation of HepG2 and mCAR-HepG2 cells after 24 h (**h**) or 48 h (**i**) with Alamar Blue assay. (See also Supplementary Figure Legends).

It has been reported that in human transformed and immortalized cells, such as hepatoma HepG2 cells, lack the capability of retaining CAR in the cytoplasm, so that CAR spontaneously accumulates in the nucleus, while in mouse hepatocytes CAR is expressed predominantly in the cytoplasm before activation^29^,^30^,^31^,^32^,^33^. In order to investigate whether CAR is involved in the effect of berberine on cell proliferation, the nucleocytoplasmic localization of CAR was evaluated in wild-type and mouse CAR stably transfected HepG2 cells (mCAR-HepG2 cells), using the confocal laser scanning microscopy (CLSM) imaging method (Fig. 1a). As expected, compared with the HepG2 cells, the green fluorescence of CAR were clearly enhanced in mCAR-HepG2 cells. Moreover, the fluorescence intensities of CAR were higher in both cytoplasma and nucleus in the mCAR-HepG2 cells than that in the HepG2 cells (Fig. 1b).

Berberine exhibits disconnection between its excellent pharmacodynamics and poor pharmacokinetic properties both *in vitro* and *in vivo*^34^,^35^. Moreover, the main methods currently used to detect berberine in plasma concentration and tissues disposition are high performance liquid chromatography (HPLC) and liquid chromatography tandem mass spectrum (LC-MS)^36^,^37^,^38^,^39^. To better understand berberine’s antiproliferation activity in hepatoma cells, we investigated the cellular uptake, distribution and localization of berberine utilizing its fluorescent molecular properties^40^,^41^, by both of flow cytometry and CLSM methods. As shown in Fig. 1c, the total cellular uptake of berberine was significantly increased in a concentration-dependent pattern (5, 10 and 25 μM), detected by the flow cytometer for 24 h treatment. However, the differences in cellular uptake of berberine were not observed between HepG2 cells and mCAR-HepG2 cells (Fig. 1d), suggesting CAR does not influence the uptake or efflux of berberine in HepG2 cells.

Furthermore, the intracellular distribution of berberine was observed in both HepG2 cells and mCAR-HepG2 cells by CLSM (Fig. 1e). Compared with control groups (DMSO), yellow fluorescent was detected in cytoplasm at the lower incubation concentration (1 μM). Under the 5 μM condition, distribution of berberine was more clearly observed in cytoplasm and around nucleus. At a higher concentration (25 μM), more fluorescence of berberine was determined in both of cytoplasm and nucleus. These results indicated that berberine was able to be cellular uptake even under lower concentration, then the intracellular distribution of berberine was gradually accumulated and located from cytoplasm into nucleus, and reached chromatin by the concentration-dependent manner in the range of 1 μM to 25 μM in HepG2 cells (Fig. 1f) and mCAR-HepG2 cells (Fig. 1g). The results also suggested that berberine might have selective impact on the proliferation of HepG2 cells and mCAR-HepG2 cells.

Effects of berberine on the cells growth and viability were evaluated by alamar blue assay. After co-incubation with different concentrations of berberine for 24 h (Fig. 1h) or 48 h (Fig. 1i), the significant differences of cell viabilities were not observed at lower concentrations of berberine (from 0.1 μM to 20 μM) in both HepG2 cells and mCAR-HepG2 cells. However, in the presence of higher concentration (50 and 100 μM), both the HepG2 cells and mCAR-HepG2 cells viabilities were significantly inhibited after incubation for 24 h and 48 h. Moreover, the cell proliferation was more significantly inhibited by berberine in mCAR-HepG2 cells than that in HepG2 cells after both 24 h and 48 h treatment (*P* < 0.01). These results supported the previous observation about berberine had higher affinity with nucleus and chromatin^42^,^43^, and suggested that the endonuclear DNA might be a target of anti-proliferating pharmacodynamics induced by berberine.

### Berberine induces apoptosis, cell cycle arrest and ROS production in absence or presence of mCAR in HepG2 cells

**Figure 2:**
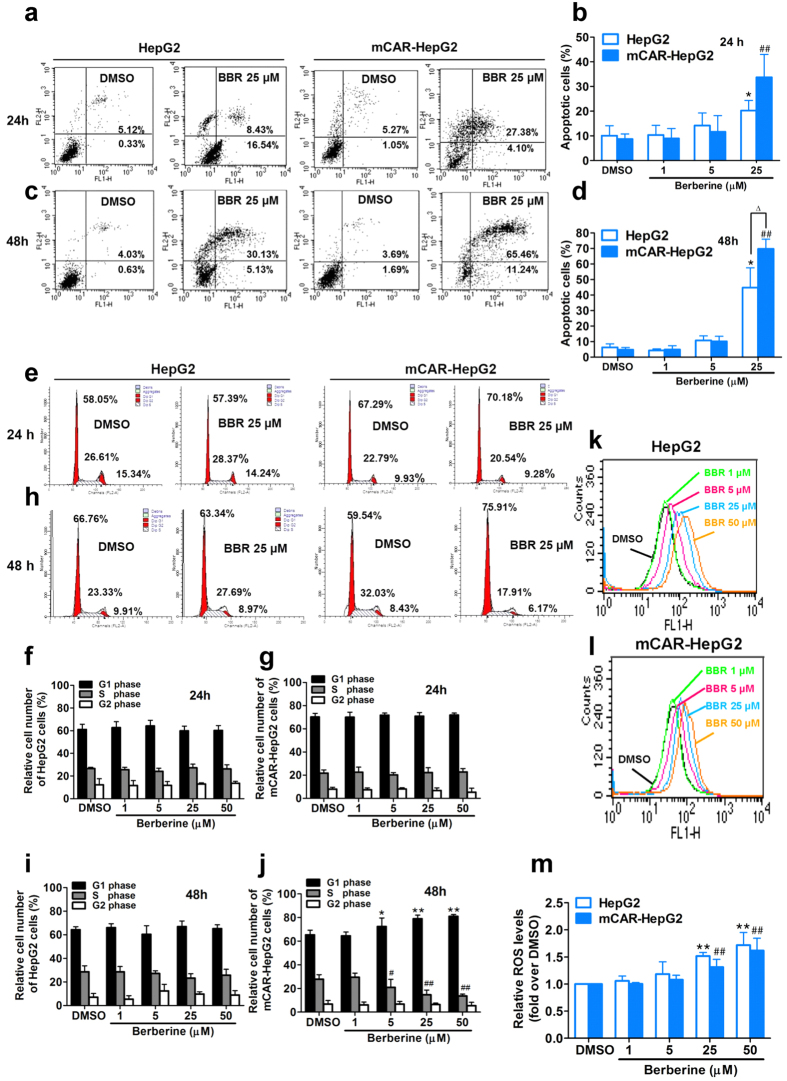
Berberine induced apoptosis, cell cycle arrest and ROS production in absence or presence of mCAR in HepG2 cells. (**a–d**): Berberine (BBR, 1, 5, 25 μM) induced apoptosis including early apoptosis (lower right quadrant) and necrosis (upper right quadrant) in HepG2 and mCAR-HepG2 cells for 24 h (**a,b**) and 48 h (**c,d**) by flow cytometry. (**e–j**): Effect of berberine (BBR, 1, 5, 25, 50 μM) on cell cycle distribution of HepG2 and mCAR-HepG2 cells for 24 h (**e–g**) and 48 h (**h–j**). (**k–m**): Berberine (BBR, 1, 5, 25, 50 μM) induced reactive oxygen species (ROS) production in HepG2 cells (**k,m**) or mCAR-HepG2 cells (**l,m**) for 24 h. (See also Supplementary Figure Legends).

To confirm whether inhibition of proliferation by berberine has impact on cancer cell survival, the percentage of apoptotic cells were counted with annexin V/propidium iodide (PI) double fluorescent staining by flow cytometry. The total apoptosis including early apoptotic cells were shown in the lower right quadrant of the scatter plot and necrotic cells in the upper right quadrant. Berberine induced the apoptosis in a dose- and time- dependent manner in the range of 1, 5 and 25 μM after treated with berberine for 24 h (Fig. 2a,b) and 48 h (Fig. 2c,d). Similar to the results of cell viability/proliferation, the rates of the apoptosis induced by berberine in mCAR-HepG2 cells were significantly higher than that in the HepG2 cells. These results are consistent with previous reports that berberine induces apoptosis in multiple human cancer cells^44^,^45^, suggesting that the inhibition of proliferation by berberine might be mediated by induction of apoptosis.

Furthermore, the effects of berberine on the cell cycle progression were detected using prodium iodide (PI) probes and analyzed with flow cytometry. Compared with 24 h time point (Fig. 2e–g), berberine promoted cell cycle arrest at G0/G1 phase, conversely, the number of cells in the S phase were decreased accordingly by berberine in the range of 1, 5, 25, 50 μM concentrations after treatment of 48 h (Fig. 2h–j). Similar to the above results of antiproliferation and inducing apoptosis, berberine-enhanced percentage of cell cycle arrest at G0/G1 phase were higher in mCAR-HepG2 cells than that in HepG2 cells, suggesting that the inhibitory effects of berberine on the mCAR-HepG2 cells proliferation were more sensitivity than that in HepG2 cells.

Reactive oxygen species (ROS) has been reported to mediate oxidative damage in berberine induced apoptosis, necrosis and inhibition of proliferation in several types of tumor cells^46^,^47^. Therefore, the present research investigated whether berberine induced more ROS production in mCAR-HepG2 than that in HepG2 cells. The ROS production was determined using dihydroethidium, as a cellular fluorescence dye analyzed by flow cytometry. The present results showed that berberine increased ROS production in the range of 1, 5, 25, 50 μM at concentration-dependent manner in both HepG2 cells (Fig. 2k) and mCAR-HepG2 cells (Fig. 2l). Unexpectedly, berberine-induced ROS production in mCAR-HepG2 cells was less than that in HepG2 cells, suggesting that there might be both ROS-dependent and ROS-independent mechanisms of mCAR-HepG2 cells death stimulated by berberine (Fig. 2m). Because the cytotoxicity-induced by ROS is also recognized as one of the cause of side effects of anti-tumor chemotherapy^48^, the ROS-independent mechanism induced by berberine might be more beneficial for chemotherapy in clinic.

### Berberine inhibits intracellular accumulation of CAR and suppresses expressions of *CYP2B6* and *CYP3A4* mRNA

**Figure 3:**
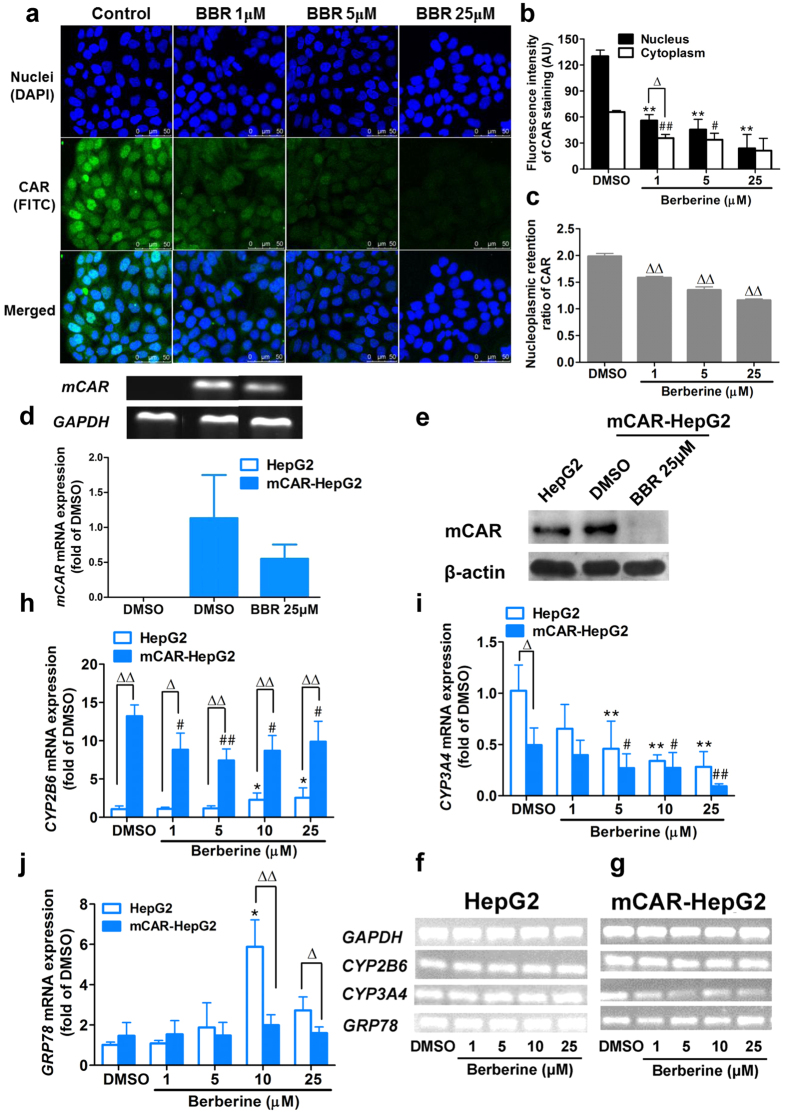
Berberine inhibited intracellular accumulation of CAR and expressions of *CYP2B6* and *CYP3A4* mRNA. (**a–e**): Visual inhibition (**a**) and quantitative analysis of berberine (BBR, 1, 5, 25 μM) on the intracellular distribution (**b**), nucleoplasmic ratio (**c**), mRNA (**d**) and protein expression (**e**) of CAR in mCAR-HepG2 cells after 24 h detected by CLSM. (**f–j**): Effects of berberine on expressions of *CYP2B6* (**h**) and *CYP3A4* (**i**) and *GRP78* (**j**) mRNA in HepG2 (**f**) and mCAR-HepG2 (**g**) cells for 24 h treatment. (See also Supplementary Figure Legends).

In our previous study, the effect of berberine on the early phase of hepatocarcinogenesis stimulated by diethylnitrosamine (DEN) plus phenobarbital (PB) in rats was investigated *in vivo*. We found that oral administration of berberine (50 mg/kg) alleviated hepatomegaly, inhibited the hepatocyte proliferating cell nuclear antigen (PCNA) expressions, decreased cytochrome P450 content and inhibited CYP2E1 and CYP1A2 activities in DEN-plus-PB treated rats^49^. Moreover, several researchers also reported that berberine inhibited the expression and activity of CYP450 enzymes *in vivo* and *in vitro*^50^. The present study further investigated whether berberine affects the upstream transcription factors on CYP450 expression, especially nuclear receptor CAR, in the subcellular location determined with CLSM in the mCAR-HepG2 cells. The present result showed that berberine significantly inhibited the accumulation of CAR protein (Fig. 3a,b), the nucleoplasmic retention ratios (Fig. 3c), expression of CAR mRNA (Fig. 3d) and protein (Fig. 3e) in mCAR-HepG2 cells in the range of 1 to 25 μM at a concentration-dependent manner.

In the absence of berberine (DMSO group), a difference in expression of *CYP3A4, CYP2B6* and *glucose regulated protein 78* (*GRP78*) genes were observed between HepG2 (Fig. 3f) and mCAR-HepG2 cells (Fig. 3g). The expressive level of *CYP2B6* gene was higher than that of *GRP78* and *CP3A4* in mCAR-HepG2 cells, whereas *CYP3A4* and *GRP78* were higher in HepG2 cells. Compared with control (DMSO), berberine markedly inhibited the expression of *CYP2B6* mRNA in mCAR-HepG2 cells (*P* < 0.05). However, berberine seemed to enhance the expression of *CYP2B6* mRNA in HepG2 cells (Fig. 3h). Moreover, berberine inhibited expression of *CYP3A4* in mCAR-HepG2 and HepG2 cells in the dose-dependent manner (1, 5, 25 μM) (Fig. 3i). Similar to the berberine effect on ROS production described above, the present result showed that berberine-enhanced expression levels of *GRP78* mRNA (Fig. 3j) in mCAR-HepG2 cells were lower than that in HepG2 cells (*P* < 0.05). GRP78 is not only considered as one of chaperones with CAR-shuttling between cytoplasm and nucleus, it is also accepted as one of the biomarkers in endoplasmic reticulum stress^51^. Therefore, this result further supported that the non-oxidative stress dependent mechanism likely involve in the berberine induced inhibition of proliferation in mCAR-HepG2 cells.

### Berberine alters DNA methylation status of whole genome and promoter region CpG sites in *CYP2B6* and *CYP3A4* genes

**Figure 4:**
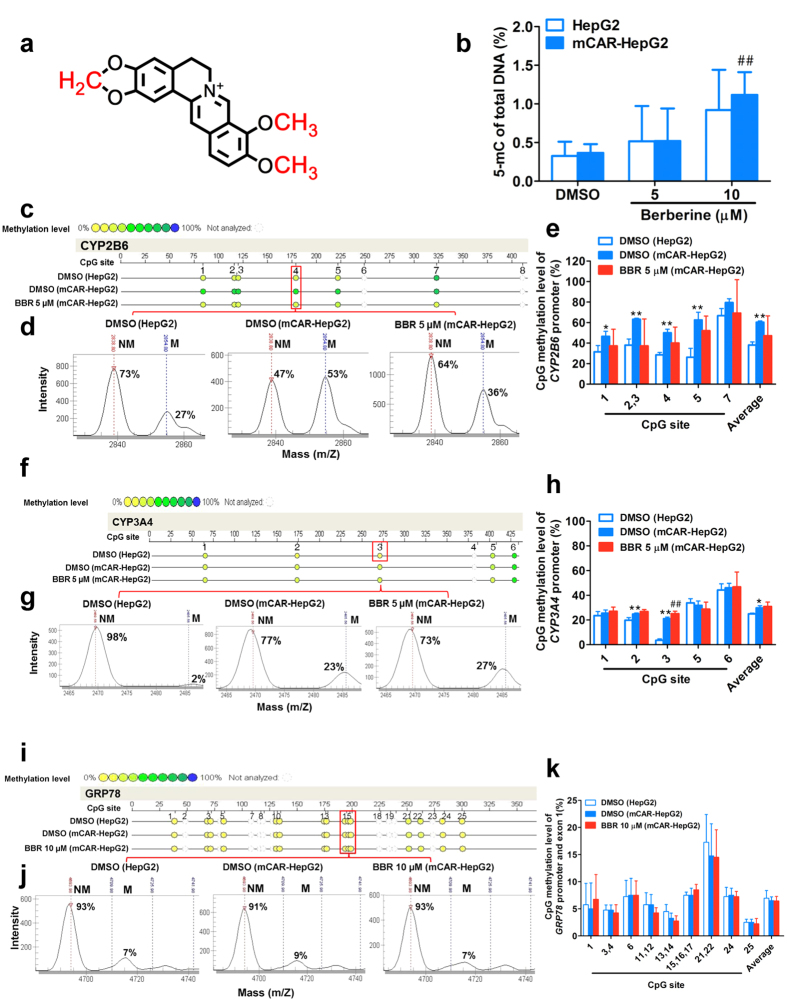
Berberine altered DNA methylation status of global genome and CpG sites within promoter regions in *CYP2B6*, *CYP3A4* and *GRP78* genes. (**a**) The three major demethylation metabolic sites on molecular structure of berberine. (**b**) Berberine enhanced the levels of global genomic DNA methylation in HepG2 and mCAR-HepG2 cells. (**c–e**): Methylation levels of *CYP2B6* gene proximal promoter region under absence or presence of berberine in HepG2 and mCAR-HepG2 cells measured by MALDI-TOF-MS method. Profiling (**c**), representative mass spectral signal patterns (**d**), individual and average levels of eight CpG site-specific methylation (**e**). (**f–h**): Methylation levels of *CYP3A4* gene promoter region. Profiling (**f**), representative mass spectral patterns (**g**), six CpG site-specific methylation (**h**). (**i–k**): Methylation levels of *GRP78* gene promoter region. Profiling (**i**), representative mass spectral patterns (**j**), 25 CpG site-specific methylation (**k**). (See also Supplementary Figure Legends).

As shown in Fig. 4a, there are three major demethylation metabolic sites have been confirmed in the molecular structure of berberine^52^. Then, this research investigated whether the inhibition of berberine on the gene expression is associated with the methylation status change of CAR target gene. As shown in Fig. 4b, the global DNA methylation levels were enhanced by berberine in the range from 5 to 25 μM, in both of HepG2 and mCAR-HepG2 cells. These results supported the hypothesis that demethylated metabolism of berberine is likely to cause the genomic DNA methylation, hence enhances the global methylation level and increase the chromosomal stability of metabolic enzymes^20^.

It has been reported that the methylation of CpG residues in promoter region might be provided the molecular docking sites, and sequentially recruited transcription factor to modify chromatin and regulate gene expression^53^. Thus, the methylation levels of total eight individual and average CpG sites (5-methylation of cytosine basic group) in *CYP2B6* proximal promoter region were analyzed by matrix-assisted laser desorption ionization time of flight mass spectrometry (MALDI-TOF-MS) method. As shown in Fig. 4c,d, the methylation levels of every individual and average CpG sites in *CYP2B6* gene promoter in mCAR-HepG2 cells, were significantly higher than that in HepG2 cells, except at the 6 th and 8 th CpG sites for which the signal were not detected (*P* < 0.05). This result supported that CAR induce the hypermethylation in *CYP2B6* promoter region and leads for inhibition of transcription of anti-oncogenes, then causing tumor cellular proliferation^54^. On the other hand, berberine significantly reduced the hypermethylation status of individual and average CpG sites in *CYP2B6* promoter in mCAR-HepG2 cells (*P* < 0.05) (Fig. 4e), suggesting that the epigenetic modifying of berberine on the *CYP2B6* promoter might be an early event and likely involved in the inhibition mechanism of *CYP2B6* gene expression in mCAR-HepG2 cells.

Similar to the effect of berberine on *CYP2B6* gene, the methylation levels of two individual CpG sites (CpG 2nd and CpG 3rd sites) and average CpG sites within *CYP3A4* gene promoter region in mCAR-HepG2 cells were higher than that in HepG2 cells (Fig. 4f,g). However, the significantly decrease of average methylation level induced by berberine (5 μM) were not observed in all CpG sites within *CYP3A4* gene promoter region (Fig. 4h). These results suggested that inhibition mechanism of berberine on *CYP3A4* gene expression might be involved in enhancing methylation level of global genome, rather than the hypermethylation in the promoter region induced by CAR in HepG2 cells. Furthermore, regardless of absence or presence of berberine (10 μM), the significantly difference were not observed in methylation levels of individual and average CpG sites within *GRP78* gene promoter region between HepG2 and mCAR-HepG2 cells (Fig. 4i–k).

## Conclusion

**Figure 5:**
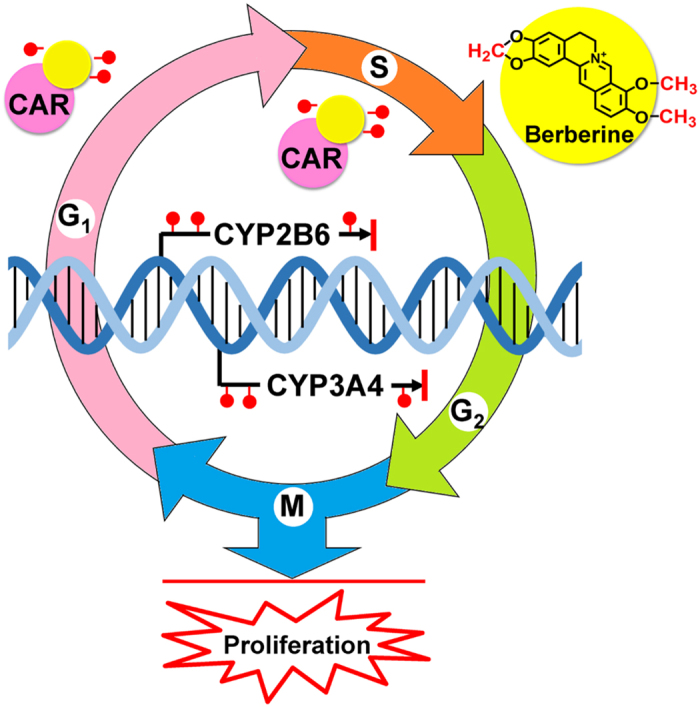
The schematic diagram represents the proliferation of berberine mediated by epigenetic modification of constitutive androstane receptor (CAR) metabolic pathway in hepatoma cells. Berberine (BBR) is accessible to nuclear chromatin, alters DNA methylation states, suppresses expressions of constitutive androstane receptor (CAR) and its target genes *cytochrome P450 2B6* (*CYP2B6*) and *CYP3A4*, arrests cell cycle and inhibits proliferation in hepatoma cells.

Our research showed that berberine is able to be cellular uptake and accessible to chromatin in human hepatoma HepG2 cells. Berberine induces more apoptosis, cell cycle arrest, but less ROS production in mCAR-HepG2 cells than that in HepG2 cells. Moreover, berberine reduced intracellular accumulation of CAR, suppressed gene expression of CYP2B6 and CYP3A4 metabolic enzymes. Furthermore, berberine enhances DNA methylation level in whole genome but reduces that in promoter regions of *CYP2B6* and *CYP3A4* genes under the presence of CAR condition. These results indicated that the anti-proliferative effect of berberine might be mediated, at least partially by the unique epigenetic modifying mechanism of CAR metabolic pathway, suggesting that berberine is a promising candidate drug in anticancer adjuvant chemotherapy, with its distinct pharmacological properties and already demonstrated safety in clinic (Fig. 5).

## Materials and Methods

A full explanation of all methods used can be found within the Supplemental Information.

### Plasmid, stable transfection and cell culture condition

Human hepatoma HepG2 cells were obtained from the American Type Culture Collection (ATCC, USA). The plasmid pCR3-mCAR expressing mouse CAR cDNA was constructed and kindly provided by Professor Hong-Bing Wang (Department of Pharmaceutical Sciences, School of Pharmacy, University of Maryland, USA)^55^,^56^. Plasmids were stably transfected into HepG2 cells (mCAR-HepG2 cell line) by Lipofectamine^TM^ 2000 according to the manufacturer’s guidelines. (See also supplementary materials and methods).

### Confocal laser scanning microscopy (CLSM) imaging

Both intracellular distribution of berberine utilizing its fluorescent molecular properties, and subcellular location of constitutive androstane receptor (CAR) were determined by confocal laser scanning microscopy (CLSM, Leica TCS SP5, Heidelberg, Germany) imaging method.

### Flow cytometry

Detections of cell cycle, apoptosis, reactive oxygen species (ROS) and intracellular distribution of berberine were performed using BD FACS Calibur flow cytometer (BD Biosciences, Franklin Lakes, NJ, USA).

### Assessment of cell viability/proliferation

Assessment of cell viability/proliferation was performed by commercial Alamar Blue kit obtained from SunBio Co. (Shanghai, China) at 570 nm and 620 nm (OD_570_-OD_620_) by a spectrophotometer Multiskan MK3 (Thermo Fisher Scientific, USA).

### RNA extraction and quantitative real-time PCR (qRT-PCR)

Total RNA was isolated from cultured cells using RNApure kit (BioTeke, Beijing, China) according to the manufacturer’s instructions. RNA purity was determined from the ratio of optical density value (OD260/280) in range of 1.8–2.1. Quantitative real-time polymerase chain reaction (qRT-PCR) was performed with a Stratagene Mx3005P QPCR system (Agilent, Biosystems, Forest City, CA, USA). (See also supplementary materials and methods).

### DNA extraction and global DNA methylation analyses

Genomic DNA was extracted from cultured cells with NucleoSpin Tissue kit (MACHEREY-NAGEL, Germany) according to manufacturer’s instructions. Global genomic DNA methylation was detected with the Methylamp Global DNA Methylation Quantification Ultra Kit (Epigentek, New York, NY) according to manufacturer’s instructions. The methylated fractions of DNA are recognized by an anti-5-methylcytosine antibody and quantified by an enzyme-linked immunosorbent assay-like reaction.

### Methylation analysis in promoter region by MassARRAY platform

Specific DNA methylation status of *CYP2B6*, *CYP3A4* and *GRP78* promoter was estimated via the Sequenom MassARRAY platform (CapitalBio, Beijing, China). The procedure of quantitative DNA methylation analysis of *CYP2B6*, *CYP3A4* and *GRP78* genes in promoter regions included bisulfite treatment of DNA, PCR amplification, *in vitro* transcription, RNA base-specific cleavage, and matrix-assisted laser desorption ionizationtime of flight mass spectrometry (MALDI-TOF-MS) analysis (MassARRAY Analyzer 4 system, Sequenom, San Diego, CA, USA)^57^,^58^. (See also supplementary materials and methods).

### Statistical analysis

Results are presented as mean ± standard deviation (SD) and analyzed by SPSS software (version 16.0). Statistical significance of mean values between multiple treatment groups was accessed by one-way analysis of variance (ANOVA). *P* value < 0.05 was considered statistically significant.

## Additional Information

**How to cite this article**: Zhang, L. *et al.* Antiproliferation of berberine is mediated by epigenetic modification of constitutive androstane receptor (CAR) metabolic pathway in hepatoma cells. *Sci. Rep.* **6**, 28116; doi: 10.1038/srep28116 (2016).

## Supplementary Material

Supplementary Information
