# Supplementary material for: Antiproliferation of berberine is mediated by epigenetic modification of constitutive androstane receptor (CAR) metabolic pathway in hepatoma cells
Source: Sci Rep. 2016 Jun 17;6:28116. doi: 10.1038/srep28116 (PMC4911599; doi:10.1038/srep28116)
Supplement: Supplementary Information [file srep28116-s1.pdf]

Antiproliferation of berberine is mediated by epigenetic modification of constitutive androstane receptor (CAR) metabolic pathway in hepatoma cells

Lei Zhang[1]*, Xiao-Jie Miao[1]*, Xin Wang[1], Hai-Hui Pan[1], Pu Li[1], Hong Ren[1], Yong-Rui Jia[2], Chuang Lu[3], Hong-Bing Wang[4], Lan Yuan[2] and Guo-Liang Zhang[1]

Supplemental Information

Supplemental Materials and Methods

1. Chemical reagents and biological materials

Berberine and geneticin (G418) were purchased from Sigma-Aldrich (St. Louis, MO, USA), Dulbecco's modified Eagle's medium (DMEM) and Lipofectamine^{TM} 2000 were purchased from Invitrogen (Carlsbad, CA, USA), and fetal bovine serum (FBS) was from Genetimes Technology (Shanghai, China). Fluorescent dye Hoechst 33258 was purchased from Molecular Probes Inc. (Oregon, USA). All the other reagents were acquired from standard commercial sources.

2. Plasmid, stable transfection, cell culture and treatment

Human hepatoma HepG2 cells were obtained from the American Type Culture Collection (ATCC, USA). The plasmid pCR3-mCAR expressing mouse CAR cDNA was constructed and kindly provided by Professor Hong-Bing Wang, (Department of Pharmaceutical Sciences, School of Pharmacy, University of Maryland, USA)[1,2]. Plasmids were stably transfected into HepG2 cells (mCAR-HepG2 cell line) by Lipofectamine^{TM} 2000 according to the manufacturer's guidelines. Both HepG2 cells and mCAR-HepG2 cells were cultured in DMEM supplemented with 10 % fetal bovine serum. Cell cultures were maintained at 37 ℃ in humidified incubator with 5 % CO_2. The stock solution of berberine was prepared in dimethylsulfoxide (DMSO, 100 mM). For experimental procedures, cells were seeded in 96-well plates at a density of 4×10^3 cells per well and at about 60 % confluence, the cells were treated with different concentrations of berberine by diluting in complete culture medium, or with 0.1 % DMSO (final concentration) as the vehicle control for different time (0, 24 or 48 h).

3. Evaluation of cell viability/proliferation by alamar blue assay

Assessment of cell viability/proliferation was performed by commercial Alamar Blue kit obtained from SunBio Co. (Shanghai, China). The principle of Alamar Blue assay is based on the intracellular enzymes to reduce the non-fluorescent blue dye to a pink fluorescent compound[3,4]. Thus, cell viability can be determined proportionally according to the colour changes by fluorometry or spectrophotometry. After 24 h or 48 h exposure to 100 μL culture media containing different concentrations of berberine, 10 μL of Alamar Blue dye was added to each well of the 96-well plates that were then incubated at 37 ℃ in 5 % CO_2 for further 2.5 h. After this time, Alamar Blue fluorescence was measured at 570 nm and 620 nm (OD_{570}-OD_{620}) by a spectrophotometer Multiskan MK3 (Thermo Fisher Scientific, USA). Wells containing medium and Alamar Blue without cells were set as blanks. Experiments were performed in quadruplicate.

4. Confocal laser scanning microscopy (CLSM) imaging

4-1. Intracellular location of CAR assay by confocal laser scanning microscopy (CLSM) imaging

To study the subcellular location of constitutive androstane receptor (CAR), the confocal laser scanning microscopy (CLSM, Leica TCS SP5, Heidelberg, Germany) imaging was performed with the specific CAR antibody/fluorescein isothiocyanate (FITC) fluorescence labeling method. Immunofluorescence staining was conducted according to the recommended protocol of Abcam Company (http://www.abcam.com/index.html?pageconfig=resource&rid=11459). Briefly both HepG2 cells and mCAR-HepG2 cells were seeded at a density of 2×10^4/well on 8-well glass slides (Nunc Lab-TekⅡ, Thermo Scientific). After 24 h exposures to culture media containing different

concentrations of berberine, these cells were washed with cold PBS twice and fixed immediately with 4 % paraformaldehyde in PBS for 15 minutes at room temperature. After washing twice with cold PBS, the cells were permeabilized with PBS containing 0.25 % Triton X-100 for 10 minutes at room temperature. After three rinses with PBS for 5 minutes, the cells were blocked with 1 % bovine serum albumin (BSA) in PBS/Tween 20 for 30 minutes at room temperature. The next step was incubation with the primary antibody against CAR (Santa Cruz, USA) diluted in 1 % BSA in PBS/Tween 20 (1:100) in a humidified chamber overnight at 4 ℃. After washing with PBS (three times for 5 minutes each), secondary antibody conjugated with FITC (Santa Cruz, USA) diluted in 1 % BSA in PBS/Tween 20 (1:25) was added and incubated for 1 h at room temperature in dark. The cells were rinsed three times with PBS for 5 minutes each in dark. At last, nucleus counter-staining of cells was done with 4', 6'-diamino-2-phenylindole (DAPI, 1 μg/ml) in PBS for 10 minutes[5-7]. The 488 nm fluorescence of FITC labeled CAR was detected and the microscopic images were captured by CLSM with 40 × or 100× objective. The blue fluorescence of cell nucleus was excited at 405 nm, and the emission of 440-460 nm was collected. Fluorescence intensity of CAR was quantified by the software platform of Leica Application Suite Advanced Fluorescence (LAS AF, Heidelberg, Germany).

4-2. Intracellular distribution of berberin assay by confocal laser scanning microscopy (CLSM) imaging

To study the intracellular distribution of berberine, the confocal laser scanning microscopy (CLSM, Leica TCS SP5, Heidelberg, Germany) imaging method was performed utilizing its fluorescent molecular properties. Briefly, cells were seeded and cultured at a density of 2×10^4/well on 8-well glass slides. After 24 h exposures to culture media containing different concentrations of berberine,

cells were rinsed three times with PBS, followed by staining the cell nuclei by Hoechst 33258 (5 μg/ml) in medium at 37 ℃ in 5 % CO_2 for 30 minutes. Then the imagings of cells and berberine were collected by a confocal laser scanning microscope (CLSM, Leica TCS SP5, Heidelberg, Germany)[8,9]. The 510-560 nm fluorescence of berberine excited by 405 nm laser was detected and the microscopic images were captured by CLSM with 40 × objective. The blue fluorescence of cell nucleus was excited at 405 nm, and the emission of 440-460 nm was collected. Fluorescence intensity of berberine was quantified by the software platform of Leica Application Suite Advanced Fluorescence (LAS AF, Heidelberg, Germany).

5. Flow cytometry

Detections of apoptosis, cell cycle distribution, and reactive oxygen species (ROS) production were performed using BD FACS Calibur flow cytometer (BD Biosciences, Franklin Lakes, NJ, USA). Moreover, the total cellular uptake of berberine was also detected by flow cytometry method utilizing its fluorescent molecular properties, similar to the principle of CLSM method.

5-1. Apoptosis assay by flow cytometry

Detection of cell apoptosis was performed using annexin V-fluorescein isothiocyanate (FITC)/propidium iodide (PI) double staining kit (Peking university center for human disease genomics). Briefly, about 1×10^6 cells/well in 6-well plates were digested by Trypsin-EDTA, centrifuged, washed twice with cold phosphate buffered saline (PBS) and resuspended in 200 μL binding buffer. Then 10 μl Annexin V-FITC (20 μg/mL) was added and the solution was incubated for 15 minutes at room temperature in dark place. After adding 300 μL binding buffer and 5 μL propidium iodide (PI, 50 μg/mL), the samples were analyzed by BD FACS Calibur flow cytometer

(BD Biosciences, Franklin Lakes, NJ, USA) within 1 hour. The Annexin V-FITC signals 488 nm/575 nm (excitation wavelength/emission wavelength) were collected using FL1 detector, and the PI signals 488 nm/620 nm (excitation wavelength/emission wavelength) were collected using FL2 detector. The median-late and early apoptotic cells were separately displayed in the upper right and lower right quadrants of the FACS dotplot.

5-2. Cell cycle distribution assay by flow cytometry

Detection of cell cycle distribution was performed using propidium iodide (PI) staining by BD FACS Calibur flow cytometer (BD Biosciences, Franklin Lakes, NJ, USA). Briefly, both HepG2 cells and mCAR-HepG2 cells were cultured at a density of 2×10^5 cells/well in 6-wells culture plates and treated with different concentrations of berberine for 24 h or 48 h; after the incubation period, cells were harvested by centrifugation. The harvested cells were fixed gently with 75 % ethanol (stored at −20 °C) at 4 ℃ overnight. Following centrifugation and PBS rinsing, fixed cells were incubated with RNase (10 mg/ml) in 37℃ water bath for 30 minutes. Finally cells were stained with PI (50 μg/ml) and analyzed at 488 nm/575 nm (excitation wavelength/emission wavelength) by BD FACS Calibur flow cytometer. For each sample 10,000–12,000 cell events were recorded and the results are expressed as percentage (%) of cell distribution in each phase; one image representing each concentration at 24 h or 48 h is shown in the figure. Average distribution of cells at different cell cycle stages following treatment with berberine. Values are averages of the flow cytometry data from independent experiments, each consisting of 10,000–12,000 cell events. Cell cycle distribution was determined by FACS Calibur flow cytometry as well as ModFit software.

5-3. Reactive oxygen species (ROS) assay by flow cytometry

Intracellular reactive oxygen species (ROS) levels were detected using dichlorodihydro fluorescin diacetate (DCF-DA) staining ROS assay kit (Applygen Technologies Inc, Beijing, China) according to the manufacturer's instructions, and the amount of fluorescence was measured using flow cytometry. This compound is cell-permeable and non-fluorescent itself, but it can be oxidized to green fluorescent dichlorofluorescein (DCF) by ROS within the cells and DCF is cell-impermeable. Briefly, both HepG2 cells and mCAR-HepG2 cells were cultured at a density of 2×10^5 cells/well in 6-wells culture plates and treated with different concentrations of berberine for 24 h or after the incubation period, about 10^6 cells in 6-well plates were incubated with 10 μM DCFH-DA diluted in PBS at 37 ℃ in 5 % CO_2 for 30 minutes, washed twice in PBS, trypsinized, resuspended in 1ml PBS and subjected to flow cytometer[10,11]. The fluorescence of DCF was excited at 488 nm, and the emission of 575 nm was collected. Intracellular ROS level was represented by the intensity of DCF fluorescence determined by flow cytometric analysis. For each sample 10,000–12,000 cell events were recorded and one image representing each concentration at 24 h is shown in the figure. The fold change of the amount of fluorescence was calculated by comparing that in the treated groups to the control group. Values are averages of the flow cytometer data from independent experiments, each consisting of 10,000–12,000 cell events.

5-4. Cellular uptake content of berberine assay by flow cytometry

Detection of total uptake content of intracellular berberine was performed utilizing its fluorescent molecular properties using BD FACS Calibur flow cytometer (BD Biosciences, Franklin Lakes, NJ, USA). Briefly, both HepG2 cells and mCAR-HepG2 cells were co-incubated with different concentrations of berberine for 24 h. About 10^6 berberine-treated cells in 6-well plates were harvested in 1 ml PBS and then detected by flow cytometer. The fluorescence intensity of berberine

was measured at 488/575 nm (excitation/emission) by FACS Calibur flow cytometer. Results were presented as the mean fluorescence intensity of the collected cells.

6. RNA extraction and quantitative real-time PCR (qRT-PCR)

6-1. RNA extraction and reverse transcription

Total RNA was isolated from cultured cells using RNApure kit (BioTeke, Beijing, China) according to the manufacturer's instructions, and RNA purity was determined from the ratio of optical density value (OD260/280) in range of 1.8-2.1. Meanwhile, cDNA production efficiency was tested by *GAPDH* (phosphoglyceraldehyde dehydrogenase) amplification. RNA (2.5 μg). RNA was reverse transcribed with random hexamer primer using RevertAid™ First Strand cDNA synthesis kit (Thermo). Synthetic cDNA was diluted to a concentration that amounts to 12.5 ng/μl RNA and then used as a template for quantitative PCR.

6-2. Quantitative real-time polymerase chain reaction (qRT-PCR)

Quantitative real-time polymerase chain reaction (qRT-PCR) was performed with a Stratagene Mx3005P QPCR system (Agilent, Biosystems, Forest City, CA, USA). The primers sequences were designed using Primer Express software 5.0 (*GAPDH*), or based on the Website: http://pga.mgh.harvard.edu/primerbank (*CYP3A4* and *mCAR*). Moreover, according to previous references, the primers sequences of *CYP2B6*[12] and *GRP78*[13] were designed, respectively. The PCR program were as follows: pre-denaturation at 95 ℃ for 10 min, denaturation at 94 ℃ for 20 s, annealing at 60 ℃ for 35 s, extending at 72 ℃ for 30 s, repetition for 40 cycles, Dissociation curve analyses were performed after PCR amplification to determine the specificity of amplification products collect and analysis fluorescence signal of SYBR Green Ⅰ dye. Duplicate tests were

performed on each sample. All samples were normalized against housekeeping gene *GAPDH* using the comparative Ct (Cycle threshold) value ($2^{-\Delta\Delta Ct}$) method. ΔCt= targeting gene Ct value – housekeeping gene (internal reference gene) Ct value; ΔΔCt= measuring sample Ct value – control sample Ct value.

The detailed information regarding primers, conditions and products of PCR amplification were listed in Table S 1 as the part of Supplementary Material.

7. DNA methylation analyses

The methylation levels of genomic DNA and promoter regions of three different genes including *CYP2B6*, *CYP3A4* and *GRP78* were analyzed using the enzyme-linked immunosorbent assay and matrix-assisted laser desorption/ionization time-of-flight mass spectrometry (MALDI-TOF-MS) methods, respectively, as described in previous publications[14,15].

7-1. DNA extraction

Genomic DNA was extracted from cultured cells (1×10^6 cells per sample) with NucleoSpin Tissue kit (MACHEREY-NAGEL, Germany) according to manufacturer's instructions. Briefly, The DNA purity and concentration were assessed by the spectrophotometer to measure the intensity of the sample's light absorbance at 260 and 280 nm wavelengths. Samples with the value of OD260/280 ratio outside the range 1.7-1.9 were not used. The DNA concentration was assessed by measuring absorbance at 260 nm.

7-2. Global genomic methylation analyses

Global genomic DNA methylation was detected with the MethylFlash Global DNA Methylation

Quantification Ultra Kit (Epigentek, New York, USA) according to manufacturer's instructions. In this assay, the methylated fractions of DNA are recognized by an anti–5-methylcytosine antibody and quantified by using an enzyme-linked immunosorbent assay–like reaction. Briefly, 100 ng purified genomic DNA was added to strip-well ELISA microplate, where the methylated fraction of DNA is captured by anti-5-methylcytosine (5-mC) antibodies and assessed colorimetrically by reading the optical density (OD) at 450nm with a spectrophotometer Multiskan MK3 (Thermo Fisher Scientific, USA). The amout of methylated DNA is proportional to the OD value measured and the percentage of methylated DNA in total DNA was calculated based on the following formula:

$$5\text{-mC}\ \% = [(\text{Sample OD} - \text{Negative Control OD}) / (\text{Slope} \times 2 \times \text{Input DNA Amount})] \times 100\ \%$$

The negative control is synthetic unmethylated DNA that contains 50 % of cytosine. The positive control is methylated DNA that contains 50 % of 5-mc. The slope (OD/ng) of the standard curve (4 concentration points of positive control including 0 point) is calculated using linear regression. Input DNA amount is 100 ng.

7-3. Methylation analysis in promoter region by MassARRAY platform

Specific DNA methylation status of *CYP2B6*, *CYP3A4* and *GRP78* promoter was estimated via the Sequenom MassARRAY platform (CapitalBio, Beijing, China). Sequenom's MassARRAY platform (Sequenom, San Diego, CA, USA) was used for quantitative DNA methylation analysis of *CYP2B6*, *CYP3A4* and *GRP78* genes in promoter regions. The protocol and quality control were conducted as described in previous publications[14-17]. Briefly, the procedure included bisulfite treatment of DNA, PCR amplification, in vitro transcription, RNA base-specific cleavage, and matrix-assisted laser desorption ionizationtime of flight mass spectrometry (MALDI-TOF-MS) analysis (MassARRAY

Analyzer 4 system, Sequenom, San Diego, CA, USA). This procedure utilizes the difference in weight between nucleotides, which create a detectable pattern and thereby can be used to determine the methylation status of individual CpG units. The methylation percentage for each sample was determined by comparing the methylated peaks against the total of methylated plus unmethylated peaks using the Epityper software version 1.0 (Sequenom, San Diego, CA).

As shown in Figure S 1a, Primers for *CYP2B6* gene were selected for the promoter area 1,000 base pairs (bp) from transcription starting site (TSS) upstream (from -854 bp to -367 bp) and covered 8 CpG sites that were divided into 7 CpG units, which were designed on the basis of the reverse complemented strand as following: Forward: 5’-aggaagagagTGTAGTGGTGTAATTTTGGTTTATTG-3’ and Reverse:5’-cagtaatacgactcactatagggagaaggctCATTTATCCATACCTACTTACATCCC-3’, except for the 6th CpG site that had no signal.

Primers for *CYP3A4* gene were within the promoter region 2,000 base pairs (bp) from transcription starting site (TSS) upstream (from -1759 bp to -1270 bp) and covered 6 CpG sites, which were designed on the basis of the reverse complemented strand as following:
Forward:5’-aggaagagagTTTTTTTGGTTTGATGTTTGTTGTT-3’ and
Reverse:5’-cagtaatacgactcactatagggagaaggctCACTCACACCCAAATTCTATATCTACC-3’(Figure S 1b). The signals at the 4th CpG site was not determinated.

Primers for *GRP78* gene were within the proximal promoter and first exon region (from -131 bp to +220bp) and covered 25 CpG sites that were divided into 20 CpG units, which were designed on

the basis of the reverse complemented strand as following: Forward:5’-aggaagagagGTTAGTTTGGTGGTTTGGGTTAAT-3’ and Reverse:5’-cagtaatacgactcactatagggagaaggctCCAAAAAAAACTTCATCTTACCAAC-3’, except for the 2th, 5 th, 7 th, 8 th, 9 th, 10 th, 18 th, 19 th, 20 th, 23 th CpG sites (total 10 CpG sites) that had no signal (Figure S 1c)

The detailed information regarding primers, conditions and products of PCR amplification, and cytosine residue rates of bisulfite conversion were listed in Table S 2 as the part of Supplementary Material.

8. Statistical analysis

Results are presented as mean ± standard deviation (SD) and analyzed by SPSS software (version 16.0). Statistical significance of mean values between multiple treatment groups was accessed by one-way analysis of variance (ANOVA). Independent *t*-test (2-tailed) was performed to evaluate the difference between two groups. P value < 0.05 was considered statistically significant.

Supplementary Figure Legends (related to Figure 1)

Figure 1. Berberine is able to be celluar uptake, accessible to chromatin and inhibit proliferation in hepatoma HepG2 cells.

(a) Visual distribution of constitutive androstane receptor (CAR) in human hepatoma HepG2 cells, which were steadily transfected with mouse CAR expression plasmid (mCAR-HepG2), were observed by confocal laser scanning microscopy (CLSM).

Intracellular CAR were marked by green fluorescein isothiocyanate (FITC) and nuclei were stained by blue 4,6-diamidino-2-phenylindole (DAPI) dye. The images are same magnification (40 ×). Scale bars for these images are 100 μm. Control cells were absence of mCAR.

(b) Quantitative analysis of constitutive androstane receptor (CAR) were detected in cytoplasma and nucleus of mCAR-HepG2 and HepG2 cells by confocal laser scanning microscopy (CLSM).

$^{**}P < 0.01$, $^{\#\#}P < 0.01$, $^{\triangle\triangle}P < 0.01$, compared with nucleus, cytoplasma or total of HepG2 cells. Data shown are mean values ± SD (n=3).

(c) Intracellular uptake of autofluorescent berberine (BBR 5, 10, 25 μM) in HepG2 and mCAR-HepG2 cells were measured by flow cytometry after 24 h treatment.

The cells treated with 0.1% DMSO were used as control.

(d) Quantitative analysis of berberine (BBR) uptake in HepG2 and mCAR-HepG2 cells by

flow cytometry after 24 h treatment.

Comparison of berberine (5, 10, 25 μM) fluorescence intensities in HepG2 and mCAR-HepG2 cells after 24-h treatment (n=3).$^{*}P < 0.05$, $^{**}P < 0.01$, compared with HepG2 control; $^{\#}P < 0.05$, $^{\#\#}P < 0.01$, compared with mCAR-HepG2 control. The cells treated with 0.1% DMSO were used as control. Data shown are mean values ± SD (n=3).

(e) Visual intracellular localization of fluorescent berberine (BBR 1, 5, 25 μM) were observed by confocal laser scanning microscopy (CLSM) in HepG2 and mCAR-HepG2 cells after 24-h incubation (n=3)**.**

Yellow autofluorescent were berberine and blue nuclei were counterstained by Hoechst. Confocal images showed that yellow autofluorescent was berberine (1, 5, 25 μM) and blue nuclei were counterstained by Hoechst. The images are same magnification (40 ×). Scale bars for these images are 25 μm. Control cells were absence of BBR.

(f and g) Quantitative analysis of berberine (BBR 1, 5, 25 μM) were detected in cytoplasma and nucleus of HepG2 cells (f) and mCAR-HepG2 cells (g) after 24 h treatment by CLSM method.

(f) Comparison of berberine (1, 5, 25 μM) fluorescence intensities between cytoplasma and nucleus in HepG2 cells after 24-h treatment (n=3).

$^{*}P < 0.05$, $^{**}P < 0.01$, compared with nucleus control; $^{\#\#}P < 0.01$, compared with cytoplasma control. The cells treated with 0.1% DMSO were used as control. Data shown are mean values ± SD (n=3).

(g) Comparison of berberine (1, 5, 25 μM) accumulation between nucleus and cytoplasma

(ratio of nucleus versus cytoplasma) in HepG2 cells after 24-h treatment (n=3).

$^{*}P < 0.05$, $^{**}P < 0.01$, compared with nucleus control; $^{\#\#}P < 0.01$, compared with cytoplasma control. The cells treated with 0.1% DMSO were used as control. Data shown are mean values ± SD (n=3).

(h) Effects of berberine on mCAR-HepG2 and HepG2 cells viability in the range of concentrations from 0.1 μM to 100 μM after 24 h treatment with Alamar Blue assay.

$^{*}P < 0.05$, $^{**}P < 0.01$, compared with HepG2 control; $^{\#}P < 0.05$, $^{\#\#}P < 0.01$, compared with mCAR-HepG2 control; $^{\Delta}P < 0.05$, $^{\Delta\Delta}P < 0.01$, compared with HepG2 of same concentration of berberine treatment. The cells treated with 0.1% DMSO were used as control. Data shown are mean values ± SD (n=4).

(i) Effects of berberine on mCAR-HepG2 and HepG2 cells viability in the range of concentrations from 0.1 μM to 100 μM after 48 h treatment with Alamar Blue assay.

$^{*}P < 0.05$, $^{**}P < 0.01$, compared with HepG2 control; $^{\#}P < 0.05$, $^{\#\#}P < 0.01$, compared with mCAR-HepG2 control; $^{\Delta}P < 0.05$, $^{\Delta\Delta}P < 0.01$, compared with HepG2 of same concentration of berberine treatment. The cells treated with 0.1% DMSO were used as control. Data shown are mean values ± SD (n=4).

Supplementary Figure Legends (related to Figure 2)

Figure 2. Berberine induces apoptosis and necrosis, cell cycle arrest and ROS production in absence or presence of mCAR in HepG2 cells.

(a and c) Berberine (BBR 1, 5, 25 μM) induces apoptosis including early apoptosis (lower right quadrant) and necrosis (upper right quadrant) in HepG2 and mCAR-HepG2 cells in a dose- and time-dependent manner for 24 h (a) and 48 h (c) treatement.

Cells were treated with berberine in the range of 1, 5, 25 μM concentrations for 24 h (a) and 48 h (c), and then harvested for analysis of apoptosis. The amount of apoptosis and necrosis were detected using fluorescein isothiocyanate (FITC) and prodium iodide (PI) probes and analyzed with flow cytometry. Lower right quadrant indicated percentage of early apoptotic cells and upper right quadrant represented the nonviable, necrotic cells.

(b and d) Quantitative analysis of berberine–induced apoptosis in HepG2 and mCAR-HepG2 cells by flow cytometry after 24 h (b) and 48h (d) treatment.

$^{*}P < 0.05$, compared with HepG2 control (n=3); $^{\#}P < 0.05$, $^{\#\#}P < 0.01$, compared with mCAR-HepG2 control (n=4). The cells treated with 0.1% DMSO were used as control. Data shown are mean values ±SD.

(e) Effect of berberine (BBR, 25 μM) on cell cycle distribution of HepG2 and mCAR-HepG2 cells for 24 h treatment.

Cells were treated with berberine in the range of 1, 5, 25, 50 μM concentrations for 24 h. The cell

cycle distribution were detected using prodium iodide (PI) probes and analyzed with flow cytometry. For each sample 10,000–11,000 cell events were recorded and the results are expressed as % cell distribution in each phase; one image representing each concentration at 24 h. Average distribution of mCAR-HepG2 and HepG2 cells at different cell cycle stages following treatment with berberine. Values are averages of the flow cytometry data from independent experiments, each consisting of 10,000–11,000 cell events.

(h) Berberine (BBR, 25 μM) induces cell cycle arrest in a dose- and time-dependent manner in HepG2 cells and mCAR-HepG2 cells for 48 h treatment.

Cells were treated with berberine in the range of 1, 5, 25, 50 μM concentrations for 48 h. The cell cycle distribution were detected using prodium iodide (PI) probes and analyzed with flow cytometry. For each sample 10,000–11,000 cell events were recorded and the results are expressed as % cell distribution in each phase; one image representing each concentration at 48 h. Average distribution of mCAR-HepG2 and HepG2 cells at different cell cycle stages following treatment with berberine. Values are averages of the flow cytometry data from independent experiments, each consisting of 10,000–11,000 cell events.

(f and g) Comparison of berberine (1, 5, 25, 50 μM) on cell cycle distribution in HepG2 (f) and mCAR-HepG2 (g) cells for 24h.

Cells were treated with berberine in the range of 1, 5, 25, 50 μM concentrations for 24 h. The cells treated with 0.1% DMSO were used as control. Data shown are mean values ± SD (n=4).

(i and j) Comparison of berberine-induced cell cycle arrest in HepG2 (i) and mCAR-HepG2 (j)

cells for 48h.

Cells were treated with berberine in the range of 1, 5, 25, 50 μM concentrations for 48 h. The cells treated with 0.1% DMSO were used as control. Data shown are mean values ± SD (n=4). $^{*}P < 0.05$, $^{**}P < 0.01$, compared with G1 phase control; $^{\#}P < 0.05$, $^{\#\#}P < 0.01$, compared with S phase control; The cells treated with 0.1% DMSO were used as control. Data shown are mean values ± SD (n=4).

(k) Berberine (BBR, 1, 5, 25, 50 μM) induces reactive oxygen species (ROS) production in HepG2 cells for 24 h.

Cells were treated with berberine in the range of 1, 5, 10, 25 μM concentrations for 24 h. Intracellular ROS levels were detected using dichlorodihydrofluorescin (DHF) probe and the amount of fluorescence was measured using flow cytometry. The fold change of the amount of fluorescence was calculated by comparing that in the treated groups to the control group (0.1% DMSO).

(l) Berberine (BBR, 1, 5, 25, 50 μM) induces reactive oxygen species (ROS) production in mCAR-HepG2 cells for 24 h.

Cells were treated with berberine in the range of 1, 5, 25, 50 μM concentrations for 24 h. Intracellular ROS levels were detected using dichlorodihydrofluorescin (DHF) probe and the amount of fluorescence was measured using flow cytometry. The fold change of the amount of fluorescence was calculated by comparing that in the treated groups to the control group (0.1% DMSO).

(m) Comparison of berberine-induced reactive oxygen species (ROS) production in

mCAR-HepG2 and HepG2 cells for 24 h.

Cells were treated with berberine in the range of 1, 5, 10, 25 μM concentrations for 24 h. $^{*}P < 0.05$, $^{**}P < 0.01$, compared with HepG2 control; $^{\#}P < 0.05$, $^{\#\#}P < 0.01$, compared with mCAR-HepG2 control; The cells treated with 0.1% DMSO were used as control. Data shown are mean values ± SD (n=4).

Supplementary Figure Legends (related to Figure 3)

Figure 3. Berberine inhibits intracellular accumulation of CAR and suppresses expressions of *CYP2B6* and *CYP3A4* mRNA.

(a) Visual inhibition of berberine on the expression of constitutive androstane receptor (CAR) protein in mCAR-HepG2 cells in dose-dependent manner (1, 5, 25 μM) for 24 h treatment by confocal laser scanning microscopy (CLSM).

Intracellular CAR were marked by green fluorescein isothiocyanate (FITC) and nuclei were stained by blue 4',6-diamidino-2-phenylindole (DAPI) dye. The images are same magnification (40 ×). Scale bars for these images are 100 μm. Control cells were absence of mCAR.

(b) Berberine inhibits accumulation of constitutive androstane receptor (CAR) in cytoplasma and nucleus of mCAR-HepG2 in the dose-dependent manner (1, 5, 25 μM) for 24 h treatment.

$^{*}P < 0.05$, $^{**}P < 0.01$, compared with CAR protein in nucleus of mCAR-HepG2 cells. $^{\#}P < 0.05$, $^{\#\#}P < 0.01$, compared with CAR protein in cytoplasma of mCAR-HepG2 cells. The cells treated with 0.1% DMSO were used as control. Data shown are mean values ± SD (n=3).

(c) Effect of Berberine on the nucleoplasmic ratio of constitutive androstane receptor (CAR) in mCAR-HepG2 in the dose-dependent manner (1, 5, 25 μM) for 24 h treatment.

$^{\Delta}P < 0.05$, $^{\Delta\Delta}P < 0.01$, compared with CAR protein in mCAR-HepG2 cells (ratio of nucleus versus cytoplasma). The cells treated with 0.1% DMSO were used as control. Data shown are mean values ± SD (n=3).

(d and e) Effects of berberine on expression of *mCAR* mRNA (d) and protein (e) in 25 μM concentration (d) in mCAR-HepG2 cells for 24 h treatment by RT-qPCR and western blot methods.

Berberine inhibits mRNA and protein expression of mCAR in mCAR-HepG2 cells for 24 h treatement. The cells treated with 0.1% DMSO were used as control. Data shown are mean values ± SD (n=4).

(f and g) Effects of berberine on expression of *CYP2B6, CYP3A4* and *GRP78* mRNA in the range from 1, 5, 10, 25 μM concentration in electrophoretograms of HepG2 cells (f) and mCAR-HepG2 cells (g) for 24 h treatment by RT-qPCR method.

(h) Effects of berberine on expression of *CYP2B6* in the range of 1, 5, 25 μM concentration in HepG2 and mCAR-HepG2 cells for 24 h treatment by RT-qPCR method.

GAPDH was used as the internal control for normalization by RT-qPCR analysis. The comparative C_T ($2^{-\triangle\triangle CT}$) method was adopted to calculate fold changes in gene expression. $^{*}P < 0.05$, compared with control in HepG2 cells (n=4); $^{\#}P < 0.05$, $^{\#\#}P < 0.01$, compared with control in mCAR-HepG2 cells (n=3); $^{\triangle}P < 0.05$, $^{\triangle\triangle}P < 0.01$, compared with same concentration of berberine treatment in HepG2 cells. The cells treated with 0.1% DMSO were used as control. Data shown are mean values ± SD.

(i) Berberine inhibits expression of *CYP3A4* in HepG2 and mCAR-HepG2 cells in the dose-dependent manner (1, 5, 25 μM) for 24 h treatment.

GAPDH was used as the internal control for normalization by RT-qPCR analysis. The comparative C_T ($2^{-\triangle\triangle CT}$) method was adopted to calculate fold changes in gene expression. $^{*}P < 0.05$, $^{**}P < 0.01$, compared with control in HepG2 cells (n=4); $^{\#}P < 0.05$, $^{\#\#}P < 0.01$, compared with control in mCAR-HepG2 cells (n=3); $^{\Delta}P < 0.05$, compared with same concentration of berberine treatment in HepG2 cells. The cells treated with 0.1% DMSO were used as control. Data shown are mean values ± SD.

(j) Berberine induces expression of *glucose regulatory protein 78* (*GRP78*) in HepG2 and mCAR-HepG2 cells for 24 h treatement.

$^{*}P < 0.05$, compared with HepG2 control (n=4); $^{\Delta}P < 0.05$, $^{\Delta\Delta}P < 0.01$, compared with same concentration of berberine treatment in HepG2 cells (n=4). The cells treated with 0.1% DMSO were used as control. Data shown are mean values ± SD.

Supplementary Figure Legends (related to Figure 4)

Figure 4. Effects of berberine on DNA methylation status of whole genome and promoter regions CpG sites in *CYP2B6*, *CYP3A4* and *GRP78* genes under the presence of constitutive androstane receptor (CAR) conditions.

(a) The three major demethylation metabolic sites are highlighted as red on molecular structure of berberine.

(b) Berberine enhanced the levels of global genomic DNA methylation in the concentration-dependent manner (0, 5, 10 μM) in HepG2 and mCAR-HepG2 cells.

The levels of global DNA methylation were quantified by using an enzyme-linked immunosorbent assay like reaction. $^{\#\#}P < 0.01$, compared with mCAR-HepG2 control (DMSO, n=4).

(c) Profiling of the eight interspersed cytidine phosphate guanosine dinucleotides (CpG) site-specific methylation in *CYP2B6* gene proximal promoter region from -854 bp to -367 bp in HepG2 cells and mCAR-HepG2 cells.

Individual site-specific CpG methylation were evaluated using sodium bisulfite treatment and matrix-assisted laser desorption/ionization time-of-flight mass spectrometry (MALDI-TOF- MS) method. Each line represents a CpG methylation profile of *CYP2B6* gene proximal promoter region from -854 bp to -367 bp and location of eight interspersed CpG sites in HepG2 (DMSO, Line 1) , mCAR-HepG2 (DMSO, Line 2) and mCAR-HepG2 (5μM berberine, Line 3) cells. Colors of each circle represent the methylation level of each corresponding CpG sites (percentage of methylated

versus the sum of methylated and nonmethylated signal intensity, %). The signals at the 6 th and 8 th CpG sites were not determinated.

(d) Representative mass spectral signal patterns of individual CpG (4th) site specific methylation in *CYP2B6* gene promoter region in HepG2 (DMSO), mCAR-HepG2 (DMSO) and mCAR-HepG2 (5μM berberine) cells measured by MALDI-TOF-MS method.

The mass spectrum peaks were labeled as nonmethylated (NM, m/z was 2838.80 Da) and methylated (M, m/z was 2854.80 Da) cleavage products from the same CpG site (CpG 4th site), and both procucts were separated by 16 Da in mass due to the presence of an adenine instead of guanine by bisulfite treatment, respectively. The methylation level of individual CpG site was calculated as signal intensity of the methylated cleavage product peak (IM) divided by the sum of the signal intensities of the methylated and nonmethylated cleavage peaks (IM plus INM).

(e) The individual and average levels of CpG site-specific methylation in *CYP2B6* gene proximal promoter region (from -854 bp to -367 bp) in absence (HepG2 and mCAR-HepG2 cells) or presence of berberine (5 μM) treated mCAR-HepG2 cells.

Methylation levels of individual and average CpG sites were analyzed by matrix-assisted laser desorption/ionization time-of-flight mass spectrometry (MALDI-TOF MS) method. Eight CpG sites were numbered as site 1-8 in proximal promoter region of *CYP2B6* gene from -854 bp to -367 bp. The signals at the CpG 6 th and 8 th sites were not determinated. The mCAR-HepG2 cells: human hepatoma HepG2 cells were steadily transfected with mouse constitutive androstane receptor (mCAR) gene expression plasmid. The methylation levels of CpG sites in *CYP2B6* gene promoter in mCAR-HepG2 cells were significantly higher than that in HepG2 cells (P<0.05). Berberine (5

μM) reversed the CpG site-specific hypermethylation in *CYP2B6* gene promoter in mCAR-HepG2 cells. $^{*}P < 0.05$, $^{**}P < 0.01$, compared with HepG2 control (DMSO, n=4); $^{\#}P < 0.05$, compared with mCAR-HepG2 control (DMSO, n=4); Data are the means and standard errors.

(f) Profiling of the six interspersed CpG site-specific methylation in *CYP3A4* gene proximal promoter region from -1759 bp to -1270 bp in HepG2 cells and mCAR-HepG2 cells.

Individual site-specific CpG methylation were evaluated by sequencing with sodium bisulfite treatment and matrix-assisted laser desorption/ionization time-of-flight mass spectrometry (MALDITOF MS) method. Each line represents a CpG methylation profile of *CYP3A4* gene proximal promoter region from -1759 bp to -1270 bp and location of six interspersed CpG sites in HepG2 (DMSO, Line 1) , mCAR-HepG2 (DMSO, Line 2) and mCAR-HepG2 (5μM berberine, Line 3) cells. Colors of each circle represent the methylation level of each corresponding CpG sites (percentage of methylated versus the sum of methylated and nonmethylated signal intensity, %).

(g) Representative mass spectral signal patterns of individual CpG (3rd) site-specific methylation in *CYP3A4* gene promoter region in HepG2 (DMSO), mCAR-HepG2 (DMSO) and mCAR-HepG2 (5 μM berberine) cells measured by MADLI-TOF MS method.

The mass spectrum peaks were labeled as nonmethylated (NM, m/z was 2469.56 Da) and methylated (M, m/z was 2485.58 Da) cleavage products from the same CpG site (CpG 3rd site), and both procucts were separated by 16 Da in mass due to the presence of an adenine instead of guanine by bisulfite treatment, respectively. The methylation level of individual CpG site was calculated as signal intensity of the methylated cleavage product peak (IM) divided by the sum of the signal intensities of the methylated and nonmethylated cleavage peaks (IM plus INM).

(h) The individual and average levels of six CpG site-specific methylation in *CYP3A4* gene proximal promoter region (from -1759 bp to -1270 bp) in absence (HepG2 and mCAR-HepG2 cells) or presence of berberine (5 μM) treated mCAR-HepG2 cells.

Methylation levels of individual and average CpG sites were analyzed by matrix-assisted laser desorption/ionization time-of-flight mass spectrometry (MALDI-TOF MS) method. Six CpG sites were numbered as site 1-6 in proximal promoter region of *CYP3A4* gene from-1759 bp to -1270 bp. The signals at the CpG 4 th sites was not determinated. The mCAR-HepG2 cells: human hepatoma HepG2 cells were steadily transfected with mouse constitutive androstane receptor (mCAR) gene expression plasmid. Similar to the *CYP2B6* gene, the methylation levels of two individual CpG sites (CpG 2 and CpG3 sites) and average of all CpG sites within *CYP3A4* gene promoter region in mCAR-HepG2 cells were significantly higher than that in HepG2 cells ($P < 0.05$). However, the CpG site-specific hypermethylation in *CY3A4* gene promoter were not inhibited by berberine (5 μM) in mCAR-HepG2 cells. $^{*}P < 0.05$, $^{**}P < 0.01$, compared with HepG2 control (DMSO, n=4); $^{\#}P < 0.05$, $^{\#\#}P < 0.05$, compared with mCAR-HepG2 control (DMSO, n=4). Data are the means and standard errors.

(i) Profiling of the CpG site-specific methylation in *GRP78* gene proximal promoter and exon 1 region from -131 bp to +220 bp in HepG2 cells and mCAR-HepG2 cells.

Individual site-specific CpG methylation were evaluated by sequencing with sodium bisulfite treatment and matrix-assisted laser desorption/ionization time-of-flight mass spectrometry (MALDITOF MS) method. Each line represents a CpG methylation profile of *GRP78* gene proximal promoter and exon 1 region from -131 bp to +220 bp, which included 25 CpG sites and

were divided into 20 CpG units, in HepG2 (DMSO, Line 1) , mCAR-HepG2 (DMSO, Line 2) and mCAR-HepG2 (10 μM berberine, Line 3) cells. Colors of each circle represent the methylation level of each corresponding CpG sites (percentage of methylated versus the sum of methylated and nonmethylated signal intensity, %). The signals at the CpG sites 2, 7, 8, 15, 16, 17, 20 (total 6 CpG units) were not determinated.

(j) Representative mass spectral signal patterns of individual CpG site-specific methylation in *GRP78* gene proximal promoter and exon 1 region in HepG2 (DMSO), mCAR-HepG2 (DMSO) and mCAR-HepG2 (10 μM berberine) cells measured by MADLI-TOF MS method.

The mass spectrum peaks were labeled as nonmethylated (NM, m/z were 4693.98 and 4725.98 Da) and methylated (M, m/z were 4709.98 and 4741.98 Da) cleavage products from the same CpG sites (CpG 13th and 14th site), and both procucts were separated by 16 Da in mass due to the presence of an adenine instead of guanine by bisulfite treatment, respectively. The methylation level of individual CpG site was calculated as signal intensity of the methylated cleavage product peak (IM) divided by the sum of the signal intensities of the methylated and nonmethylated cleavage peaks (IM plus INM).

(k) The levels of CpG site-specific methylation in *GRP78* gene proximal promoter and exon 1 region (from -131 bp to +220 bp) in absence (HepG2 and mCAR-HepG2 cells) or presence of berberine (10 μM) treated mCAR-HepG2 cells.

Methylation levels of individual and average CpG sites were analyzed by matrix-assisted laser desorption/ionization time-of-flight mass spectrometry (MALDI-TOF MS) method. Twenty five CpG sites (20 CpG units) were numbered as site 1-25 in *GRP78* gene proximal promoter and exon

1 region from -131 bp to +220 bp. The signals at the CpG sites 2, 6, 7, 8, 15, 16, 17, 20 (total 6 CpG sites) were not determinated. The mCAR-HepG2 cells: human hepatoma HepG2 cells were steadily transfected with mouse constitutive androstane receptor (mCAR) gene expression plasmid._The significant differences in methylation levels of individual and average CpG sites in *GRP78* gene proximal promoter and exon 1 regione were not observed between HepG2 and mCAR-HepG2 cells in absence or presence of berberine (10 μM). $^{*}P < 0.05$, compared with HepG2 control (DMSO, n=4); $^{\#}P < 0.05$, compared with mCAR-HepG2 control (DMSO, n=4). Data are the means and standard errors.

Supplementary Figure Legends (related to Figure 5)

Figure 5. The schematic diagram represents the proliferation of berberine mediated by epigenetic modification of constitutive androstane receptor (CAR) metabolic pathway in hepatoma cells.

Berberine (BBR) is accessible to nuclear chromatin, alters DNA methylation states, suppresses expressions of constitutive androstane receptor (CAR) and its target genes *cytochrome P450 2B6* (*CYP2B6*) and *CYP3A4*, arrests cell cycle and inhibits proliferation in hepatoma cells.

Supplementary References

1. Zelko, I., Sueyoshi, T., Kawamoto, T., Moore, R. & Negishi, M. The peptide near the C terminus regulates receptor CAR nuclear translocation induced by xenochemicals in mouse liver. *Mol. Cell Biol.* **21,** 2838-2846 (2001).
2. Wang, H. *et al*. Human constitutive androstane receptor mediates induction of CYP2B6 gene expression by phenytoin. *J. Biol. Chem.* **279,** 29295-29301 (2004).
3. Hamid, R., Rotshteyn, Y., Rabadi, L., Parikh, R. & Bullock, P. Comparison of alamar blue and MTT assays for high through-put screening. *Toxicol. In Vitro.* **18,** 703-710 (2004)
4. Schoonen, W. G., Stevenson, J. C., Westerink, W. M. & Horbach, G. J. Cytotoxic effects of 109 reference compounds on rat H4IIE and human HepG2 hepatocytes. III: Mechanistic assays on oxygen consumption with MitoXpress and NAD (P) H production with Alamar Blue™. *Toxicol. In Vitro.* **26,** 511-525 (2012).
5. Hosseinpour, F. *et al*. Overexpression of the Rho-guanine nucleotide exchange factor ECT2 inhibits nuclear translocation of nuclear receptor CAR in the mouse liver. *FEBS Lett.* **581,** 4937-4942 (2007).
6. Kobayashi, K., Sueyoshi, T., Inoue, K., Moore, R. & Negishi, M. Cytoplasmic accumulation of the nuclear receptor CAR by a tetratricopeptide repeat protein in HepG2 cells. *Mol. Pharmacol.* **64,** 1069-1075 (2003).
7. Zhao, S., Yuan, L., Wang, J., Zhang, X., He, Z. & Zhang, Q. A novel and facile approach to imaging nanoparticles transport across Transwell filter grown cell monolayer in real-time and in situ under confocal laser scanning microscopy. *Biol. Pharm. Bull.* **35,** 335-345 (2012).
8. Serafim, T. L. *et al*. Different concentrations of berberine result in distinct cellular localization patterns and cell cycle effects in a melanoma cell line. *Cancer Chemother. Pharmacol.* **61,**

1007-1018 (2008).

9. Wang, G. Y., Lv, Q. H., Dong, Q., Xu, R. Z. & Dong, Q.H. Berbamine induces Fas-mediated apoptosis in human hepatocellular carcinoma HepG2 cells and inhibits its tumor growth in nude mice. *J. Asian Nat. Prod. Res.* **11,** 219-228 (2009).
10. Liu, B. *et al*. Berberine Inhibits Human Hepatoma Cell Invasion without Cytotoxicity in Healthy Hepatocytes. *PLoS One* **6,**e21416 (2011).
11. Wu, W. Y., Yang, J. Y., Du, L. M., Wu, H. & Li, C. F. Determination of ethambutol by a sensitive fluorescent probe. *Spectrochim. Acta. A. Mol. Biomol. Spectrosc.* **79,** 418-22 (2011).
12. Westerink, W. M. & Schoonen, W. G. Cytochrome P450 enzyme levels in HepG2 cells and cryopreserved primary human hepatocytes and their induction in HepG2 cells. *Toxicol. In Vitro.* **21,** 1581-1591 (2007).
13. Gu, X. *et al*. Bip overexpression, but not CHOP inhibition, attenuates fatty-acid-induced endoplasmic reticulum stress and apoptosis in HepG2 liver cells. *Life Sci.* **87,** 724-32 (2010).
14. Wu, L. et al. Altered methylation of IGF2 DMR0 is associated with neural tube defects. *Mol. Cell Biochem.* **380,** 33-42 (2013).
15. Ehrich, M. et al. Quantitative high-throughput analysis of DNA methylation patterns by base-specific cleavage and mass spectrometry. *PNAS* **102,** 15785-15790 (2005).
16. Wang, L. *et al*. Relation between hypomethylation of long interspersed nucleotide elements and risk of neural tube defects. *Am. J. Clin. Nutr.* **91,** 1359-1367 (2010).
17. Tran, S. et al. Altered methylation of the DNA repair gene MGMT is associated with neural tube defects. *J. Mol. Neurosci.* **47,** 42-51 (2012).

Appendix Table S 1 (related to Supplemental Experimental Procedures)

Appendix Table S 1 Polymerase chain reaction (PCR) amplification and analysis conditions of *CYP2B6*, *CYP3A4*, *mCAR* and g*lucose regulated protein 78* (*GRP78*) and *phosphoglyceraldehyde dehydrogenase* (*GAPDH*) genes.

Gene Name	Primer Sequences	PCR product (bp)
GAPDH	Forward:5'-GAAGGTGAAGGTCGGAGTC-3'	226
	Reverse:5'-GAAGATGGTGATGGGATTTC-3'	
CYP2B6	Forward:5'-TTAGGGAAGCGGATTTGTCTTG-3'	73
	Reverse:5'-GGAGGATGGTGGTGAAGAAGAG-3'	
CYP3A4	Forward:5'-CACGAGCAGTGTTCTCTCCTT-3'	124
	Reverse:5'-CACAGTATCATAGGTGGGTGGT-3'	
mCAR	Forward:5'-ATGGAACAACAGTCTCGGCTC-3'	107
	Reverse:5'-CGCTGAAGTTCATAGGAGTATGC-3'	
GRP78	Forward:5'-CGGGCAAAGATGTCAGGAAAG-3'	211
	Reverse:5'-TTCTGGACGGGCTTCATAGTAGAC-3'	

Appendix Table S 2 (related to Supplemental Experimental Procedures)

Appendix Table S 2 Primers, conditions and products of PCR amplification, and cytosine residue rates of bisulfite conversion in promoter region of *CYP2B6*, *CYP3A4* and *GRP78* genes.

Gene	Primer Sequences	PCR Product (bp)	Transcription Starting Site Upstream (bp)	Covered CpG Sites
CYP2B6	Forward:5’-aggaagagagTGTAGTGGTGTAATTTTGGTTTATTG-3’	226	-854 bp to -367 bp	8
	Reverse:5’-cagtaatacgactcactatagggagaaggctCATTTATCCATACCTACTTACATCCC-3’			
CYP3A4	Forward:5’-aggaagagagTTTTTTTGGTTTGATGTTTGTTGTT-3’	73	-1759 bp to -1270 bp	6
	Reverse:5’-cagtaatacgactcactatagggagaaggctCACTCACACCCAAATTCTATATCTACC-3’			
GRP78	Forward:5’-aggaagagagGTTAGTTTGGTGGTTTGGGTTAAT-3’	211	-131 bp to +220 bp	25
	Reverse:5’-cagtaatacgactcactatagggagaaggctCCAAAAAAAACTTCATCTTACCAAC-3’			

Appendix Figure S 1

a ***CYP2B6* promoter sequence (-854/-367)**

-854 ....tgcagtggtgtaatcttggctcactgcagcc
-823 tccacctcccaggttcaagtgattctcttgcctcagcctcc**cg**agtagctgggattaaaa
1
-763 gtacccaccatca**cg**cc**cg**gttaatttttgtgtttttagtacagatggggttttgccatg
2 3
-703 tgggccaggatggtct**cg**aactcttgacctcaattgatctgcccccctcagcttcccaa
4
-643 **cg**tgctgggattacaggtgtgagccac**cg**cacccagccagcctctcagttttgaacatgca
5 6
-583 ctaccaccacctccacaacacacaaatgtaaatgcacttt**cg**tatataaaactgtataaa
7
-523 tacaaggaagctcatacacatgcaaggatacacacataagcacccccagattcaaccaca
-463 gaaatata**cg**ccagtacatttgcataaattcaaacacccctttacatgtaaaaatcatat
8
-403 aagcacatacagggatgcaagcaggcatggacaaatg.......................................

b ***CYP3A4* promoter sequence (-1759/-1270)**

-1759 ....cctttttggtttgatgctt
-1740 gctgtcacttctttccttaggtgcctctctgta**cg**gctcttttatcccagggattccaga
1
-1680 gttacagcacatgcataccaccatccaagcatgtttatttgtctcctgcttcactaggct
-1620 gtccccaaggaacatgtggctcc**cg**gcacacacctggcacaacactgcacatgacattca
2
-1560 cccacttggccttgaatctgacaaggaatctggcatgatgttcacccactcaggccaggt
-1500 gc**cg**agcagccctggaggcttaggggccagagggatgggaaaaggtgtctttctggggtg
3
-1440 agtatcagtttctgcaggagggctgaatgtgagaaagaataaagagagaaggaag**cg**aac
4
-1380 aagcacagcttaaacat**cg**cctatttctattgagttttaagaa**cg**ctgtgattttgtttg
5 6
-1320 tcatgcaatccattcatcaggccaggcagacacagaacttgggtgtgagtg...................

c ***GRP78* promoter region sequence (-131/+220)**

-131 ....gccagcttggt
-120 ggcctgggccaatgaa**cg**gcctccaa**cg**agcagggccttcaccaat**cg**g**cg**gcctcca**cg**
1 2 3 4 5
-60 a**cg**gggctgggggagggtatataagc**cg**agtagg**cg**a**cg**gtgaggt**cg**a**cg**c**cg**gccaag
6 7 8 9 10 11 12
+1 acagcacagacagattgacctattggggtgttt**cgcg**agtgtgagagggaag**cg**c**cgcg**g
13 14 15 16 17
+61 cctgtatttctagacctgccctt**cg**cctggtt**cg**tgg**cg**ccttgtgaccc**cg**ggcccctg
18 19 20 21
+121 c**cg**cctgcaagt**cg**gaaattg**cg**ctgtgctcctgtgcta**cg**gcctgtggctggactgcct
22 23 24 25
+181 gctgctgcccaactggctggcaagatgaagctctccctgg...

Appendix Figure S 1 Legends

Appendix Figure S 1 (related to supplemental materials and methods)

(a) Schematic diagram of *CYP2B6* gene proximal promoter sequence from -854 bp to -367 bp and location of eight interspersed cytidine-phosphate-guanosine dinucleotides (CpG) sites. The sequence shown represents a fragment from -854 base pairs (bp) to -367 bp in the 5' untranslated region (5'-UTR) of promoter. CpG sites were numbered sequentially in these regions separately, and underlining denotes the CpG units containing more than one CpG site detected all together. Thus eight CpG sites, which were divided into seven CpG units. Polymerase chain reaction primers were designed on the basis of the reverse complemented strand of this fragment.

(b) Schematic diagram of *CYP3A4* gene proximal promoter sequence from -1759 bp to -1270 bp and location of six interspersed cytidine-phosphate-guanosine dinucleotides (CpG) sites. The sequence shown represents a fragment from -1759 base pairs (bp) to -1270 bp in the 5' untranslated region (5'-UTR) of promoter. CpG sites were numbered sequentially in these regions separately, and underlining denotes the CpG units containing more than one CpG site detected all together. Polymerase chain reaction primers were designed on the basis of the reverse complemented strand of this fragment.

(c) Schematic diagram of *glucose regulated protein 78* (*GRP78*) gene proximal promoter and exon 1 sequence from -131 bp to +220 bp and location of twenty five interspersed cytidine-phosphatte-guanonine oligodeoxynucleotides (CpG) sites. The sequence shown

represents a fragment from -131 base pairs (bp) to +220 bp in the 5’ untranslated region (5’-UTR) of proximal promoter and exon 1. CpG sites were numbered sequentially in these regions separately, and underlining denotes the CpG units containing more than one CpG site detected all together. Thus 25 CpG sites were divided into 20 CpG units. Polymerase chain reaction primers were designed on the basis of the reverse complemented strand of this fragment.
